# Anticancer Podophyllotoxin Recovery from Juniper Leaves at Atmospheric and High Pressure Using Eco-Friendly Solvents

**DOI:** 10.3390/plants12071526

**Published:** 2023-03-31

**Authors:** Diana Ivanova, Paraskev Nedialkov, Alexander Tashev, Zlatina Kokanova-Nedialkova, Marta Olech, Renata Nowak, Stanislava Boyadzhieva, George Angelov, Dragomir Yankov

**Affiliations:** 1 Institute of Chemical Engineering, Bulgarian Academy of Sciences, 1113 Sofia, Bulgaria; 2 Department of Pharmacognosy, Faculty of Pharmacy, Medical University, 1000 Sofia, Bulgaria; 3 Department of Dendrology, University of Forestry, 1756 Sofia, Bulgaria; 4 Department of Pharmaceutical Botany, Faculty of Pharmacy, Medical University of Lublin, 20-059 Lublin, Poland

**Keywords:** *Juniperus virginiana* L., podophyllotoxin, extraction optimization, supercritical fluid extraction, eco-friendly solvents, UHPLC/HRMS/MS, green chemistry, lignans

## Abstract

Podophyllotoxin (PPT) is a precursor for the synthesis of drugs against cancer and other diseases. The present sources of PPT (*Sinopodophyllum hexandrum* and *Podophyllum peltatum*) are endangered species, with PPT production highly dependent on their growing conditions. In connection with the identification of new sources of PPT, the present study aimed to recover PPT from *Juniperus virginiana* leaves via atmospheric or high pressure extraction methods with a focus on using eco-friendly solvents. PPT quantification was determined by UHPLC/HRMS/MS. A thorough study of conventional extraction was carried out to reveal the optimal conditions (solvent ethyl acetate at room temperature and a duration of 1 h) for maximizing the PPT recovery (about 30 mg/g of dry extract and 3 mg/g of dry initial plant material). Peleg’s equation was applied for process kinetics modeling. The best PPT content in the final dry extract (42–45 mg/g of dry extract) was obtained by high pressure methods under supercritical (scCO_2_ with ethanol or ethyl acetate, 30 MPa, 50 °C and 100 min) or accelerated solvent extraction conditions (solvent ethyl acetate, 10.35 MPa, 20 °C and 3 cycles for 15 min). Seasonal stability and storage stability of the raw material were also determined. The present results have potential applications in the pharmacy for the delivery of PPT from juniper leaves.

## 1. Introduction

Podophyllotoxin (PPT) is an industrial precursor for the synthesis of various drugs. PPT derivatives exhibit efficient antiviral, anthelminthic, antitumor and other biological activities with different mechanisms of action [1]. For example, etoposide, etopophos and teniposide are PPT derivatives that are known as leading standards in the treatment of various cancer diseases (lung, ovarian, testicular, bladder, stomach, pancreatic, brain, breast, blood, etc.) [2,3]. The topical agent Podofilox (containing 0.5% PPT) is used against *Condyloma acuminatum* (genital warts), which is caused by the human papillomavirus. Moreover, etoposide has been studied against the life-threatening cytokine activation caused by SARS-CoV-2 in some COVID-19 patients [4].

PPT was isolated in 1880 by Valerian Podwyssotzki from *Podophyllum peltatum* L. (American mayapple) [5]. Its structure was discovered in the 1930s by the scientists Borsche and Niemann [6]. At present, natural sources of PPT are several species of the genus *Podophyllum* L. (Berberidaceae) and *Sinopodophyllum hexandrum* (Royle) T. S. Ying (Himalayan mayapple). However, PPT biosynthesis is highly variable depending on the growing conditions [7], plant age [8], etc. In addition, American and Himalayan mayapples are already endangered species because of their intensive industrial exploitation. Recently, modern techniques for the more efficient elution of PPT from plant material were examined. Accelerated solvent extraction (ASE) was applied to reduce the extraction time at an increased pressure for the delivery of lignans from *Podophyllum hexandrum* rhizomes. ASE was performed sequentially in ethyl acetate and methanol at 50 °C and 60 °C using a 5 min heating time and 10 min static time [9]. The same authors also applied supercritical fluid extraction (SFE) with scCO_2_ for the delivery of PPT from *P. hexandrum* rhizomes using a pressure of 300 bars, a temperature of 50 °C and a process duration of 2.5 h. After that, the plant material was subjected to SFE with carbon dioxide and modified with ethyl acetate or methanol, and the extraction time was 1.5 hours. The modified solvents were found to increase the solubility of the polar PPT molecule in the supercritical fluid.

Podophyllotoxin is a widely distributed secondary metabolite in species belonging to various genera other than the genus *Podophyllum* [10,11]. The delivery of lignans, including PPT, from *Picea abies* (L.) H. Karst and other plant samples was investigated using Soxhlet’s extraction (with 95% ethanol as a better solvent) or supercritical fluid extraction with carbon dioxide containing ethanol or methanol [12].

However, efficient concentrations of PPT have been detected only in a few plant species. PPT is concentrated in juniper leaves, whereas the galbuli extracts showed negligible content of this bioactive compound. For example, the leaf extracts of several *Juniperus* representatives, including *J. virginiana* L., *J. sabina* L., *J. scopulorum* Sarg., *J. horizontalis* Moench, *J. x media*, etc., were found to exhibit high antiproliferative activities due to the efficient biosynthesis of PPT [13,14,15,16]. Junipers are evergreen plants that perform PPT biosynthesis independent of the seasonal changes throughout the year, which was determined when *J. virginiana* was used as an experimental model [17]. Therefore, junipers capable of efficient PPT biosynthesis have attracted an increasing amount of scientific interest as perspective alternative sources of PPT for use in the pharmacy.

A number of investigations have studied various extraction methods focused on the delivery of essential oils from junipers (*J. virginiana* L., *J. occidentalis* Hook, *J. ashei* J. Buchholz, etc.) using organic solvents, liquid carbon dioxide and various pressurized fluids (including subcritical water) under different pressures, temperatures and extraction times [18,19,20,21]. However, the optimal method for the most efficient elution of PPT from juniper leaves with the use of an eco-friendly approach has not yet been established.

Considering that new natural sources of PPT are necessary for use in the pharmacy, the present study was aimed at the optimization and kinetic modeling of PPT recovery from juniper leaves as an alternative source of this drug precursor. To our knowledge, this is the first optimization of PPT extraction from juniper leaves with a focus on the application of ecological solvents. The studied conditions included agitation in a shaker water bath at normal pressure, which has the advantage of being a simple and cheap technique. In addition, supercritical fluid extraction and accelerated solvent extraction methods were investigated. All analyzed methods and results are feasible for application in small enterprises or industrial producers. *J. virginiana* L., whose leaf extract was found to exhibit a high PPT content and antiproliferative activity [15,16], was chosen as an experimental model. The correlation between the experimental and theoretical results for the extraction kinetics was determined using Peleg’s modeling equation [22]. Originally, Peleg’s kinetic model was meant to describe the absorption processes. However, due to a similar asymptotic shape of the sorption and extraction kinetic curves, this modeling was widely applied for the description of the extraction curves (e.g., analysis of the extracted matter over time) of bioactive substances from plants [23,24]. PPT quantification in the juniper leaf extracts was carried out using HRMS/MS detection. The seasonal stability of the PPT biosynthesis and storage stability of the juniper leaves were also determined. The analysis showed that evergreen junipers can be used as a plant source of PPT throughout the year. As a result, optimal ecological conditions for PPT recovery from juniper leaves were studied using normal pressure, accelerated solvent extraction and supercritical extraction conditions. The present results have potential applications as environmentally friendly methods for the delivery of podophyllotoxin from juniper leaves as an alternative source of this anticancer drug precursor for use in the pharmacy.

## 2. Results

### 2.1. Extraction Optimization of PPT from Juniper Leaves at Atmospheric Pressure

The optimization of PPT extraction from juniper leaves at atmospheric pressure was carried out by varying the solvent type, temperature, liquid-to-solid ratio (LSR) and process duration. The experiments at atmospheric pressure were performed using ground plant material suspended in the corresponding solvent in a closed vessel, which was stirred in a thermostatic shaker water bath. This technique has the advantage of using simple equipment, and is applicable for the production of juniper extracts in laboratory conditions, as well as in bigger industrial reactors with agitation.

#### 2.1.1. Selection of Appropriate Solvent for PPT Extraction

PPT is a polar molecule; therefore, various polar solvents were studied for its extraction from juniper leaves, including water, methanol, ethanol, *i-*propanol, *n-*butanol, tetrahydrofuran, acetonitrile, ethyl acetate, acetone and methyl ethyl ketone. According to existing reports [17], non-polar solvents, such as petroleum ether, hexane, etc., lead to low yields of PPT extraction from juniper leaves, and thus, were not applied in the present study.

**Figure 1:**
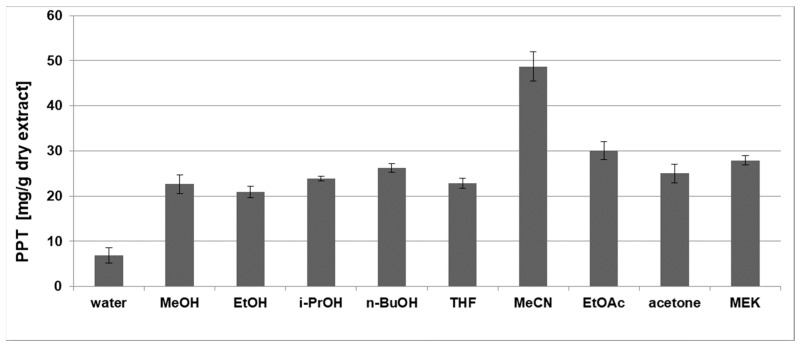
Effect of the solvent type on the PPT concentration relative to the weight of the dry juniper leaf extracts. The used polar solvents were water, methanol (MeOH), ethanol (EtOH), *i-*propanol (*i*-PrOH), *n-*butanol (*n-*BuOH), tetrahydrofuran (THF), acetonitrile (MeCN), ethyl acetate (EtOAc), acetone and methyl ethyl ketone (MEK, 2-butanone). Other extraction parameters—liquid-to-solid ratio of 10 (*v*/*w*), process duration of 5 h and room temperature.

The selection of the solvent type was carried out under the following conditions: a liquid-to-solid ratio (LSR) of 10 (*v*/*w*) and an extraction duration of 5 h at room temperature. These conditions were chosen according to our previous experience and the existing practice of establishing a long enough initial processing time in order to attain a pseudo-equilibrium state, a big enough LSR for good interphase contact and the elimination of solubility limits, and ambient temperature for avoiding the possible thermal destruction of the active component. The effect of the solvent type on the PPT recovery was analyzed according to the PPT content related to the starting dry plant material (Table 1) and to the dry extract (Figure 1).

#### 2.1.2. Determination of the Optimal PPT Extraction Temperature

**Figure 2:**
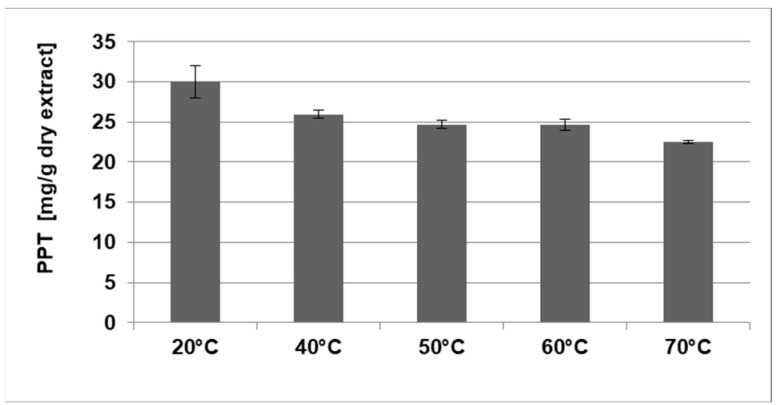
Effect of the temperature on the PPT content of dry juniper leaf extracts. Variable extraction parameter—temperature; constant parameters—solvent (ethyl acetate), liquid-to-solid ratio (10 (*v*/*w*)) and process duration (5 h).

The effect of the temperature on the PPT extraction from juniper leaves was studied at room temperature (20 °C), 40 °C, 50 °C, 60 °C and 70 °C using an extraction duration of 5 h, an LSR of 10 (*v*/*w*) and the previously selected solvent (ethyl acetate). The PPT content was measured relative to the weight of the starting plant material (Table 2), as well as relative to the weight of the final dry extract (Figure 2).

#### 2.1.3. Determination of the Liquid-to-Solid Ratio (LSR) for PPT Extraction

The LSR value for PPT extraction varied from 10 to 40 (*v*/*w*) at room temperature using an extraction duration of 5 h and the previously selected solvent ethyl acetate. An LSR less than 10 (*v*/*w*) was found to be insufficient for the collection of the extract during the filtration process and was not studied. The effect of the LSR on the PPT content was determined relative to the dry extracts and to the initial plant material (Table 3).

**Figure 3:**
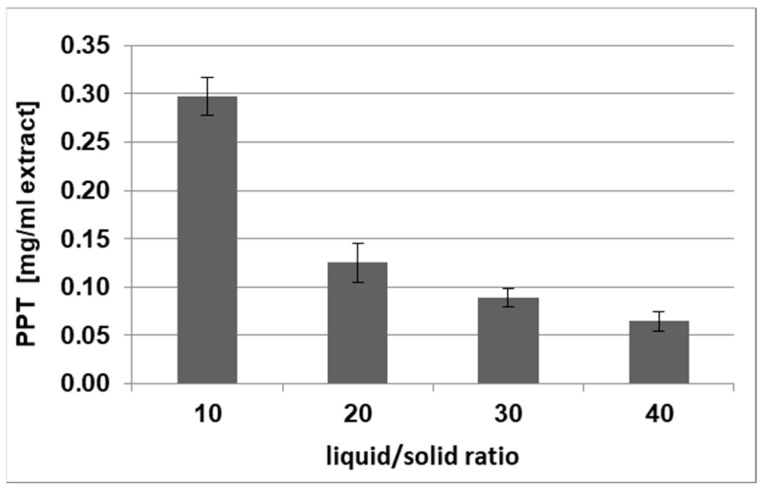
Effect of the liquid-to-solid ratio on the PPT concentration per volume of *J. virginiana* extracts. Variable parameter—liquid-to-solid ratio (LSR, *v*/*w*); constant parameters—process duration (5 h), previously selected solvent (ethyl acetate) and room temperature (20 °C).

The content of PPT was also expressed per volume of the extract in order to analyze the solvent consumption for PPT recovery (Figure 3).

#### 2.1.4. Optimization of the Extraction Time and Peleg’s Kinetic Modeling

**Figure 4:**
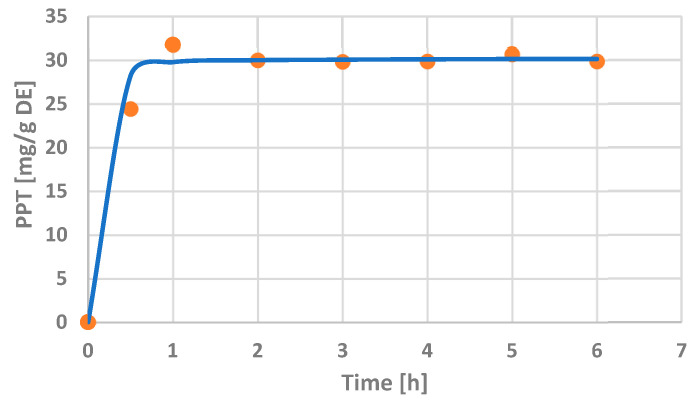
Effect of the extraction time on the PPT concentration in *J. virginiana* leaf extracts and correlation of Peleg’s equation curve (line) with the experimental data (points). Variable parameter—extraction time; constant parameters (previously selected conditions)—solvent ethyl acetate, room temperature and liquid-to-solid ratio of 10 (*v*/*w*). Abbreviations: PPT—podophyllotoxin; DE—dry extract.

The extraction time varied from 30 min to 6 h under atmospheric pressure in a shaker water bath at room temperature using the solvent (ethyl acetate) and LSR (10 *v*/*w*) selected in the previous experiments. Experimental and theoretical data were correlated by Peleg’s modeling of the extraction kinetics. Figure 4 represents the experimental data for the extraction kinetics (points) superimposed onto the predictions made with Peleg’s equation (solid line). Generally, a good match between the model and experimental results is observed. Additionally, a precise prediction of the equilibrium PPT concentration is illustrated.

**Figure 5:**
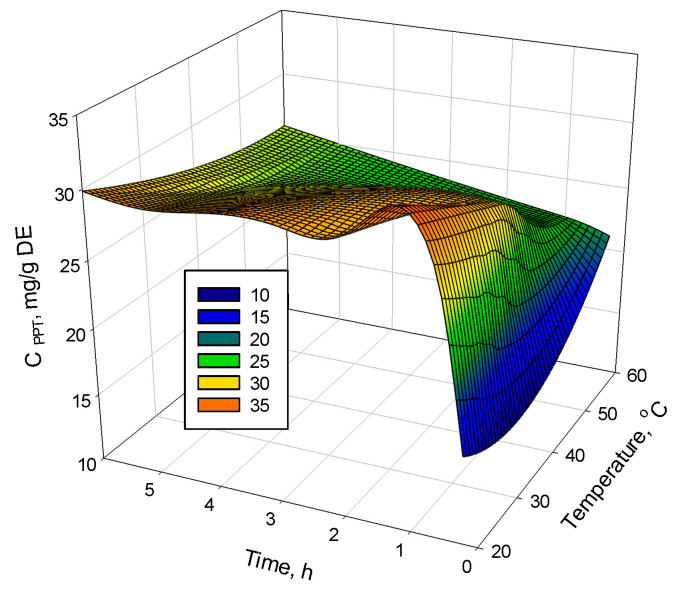
A 3D presentation of the influence of the extraction time and temperature on the PPT content (mg/g DE) in *J. virginiana* leaf extracts, obtained at normal pressure under agitation (shaker water bath).

The 3D analysis of the PPT concentration was presented as a function of the extraction temperature and time (Figure 5). The maximum PPT concentration of the obtained dry extracts at an LSR of 10 (*v*/*w*) was observed at room temperature, using solvent ethyl acetate and a 1 h extraction time.

### 2.2. Supercritical Fluid Extraction of PPT from Juniper Leaves

The plant material (dry ground juniper leaves) was subjected to supercritical fluid extraction (SFE) using neat carbon dioxide or a mixture of carbon dioxide and co-solvents (Table 4). Ethanol and ethyl acetate were applied as co-solvents in order to change the solvent polarity, which was expected to improve the solubility of PPT.

**Figure 6:**
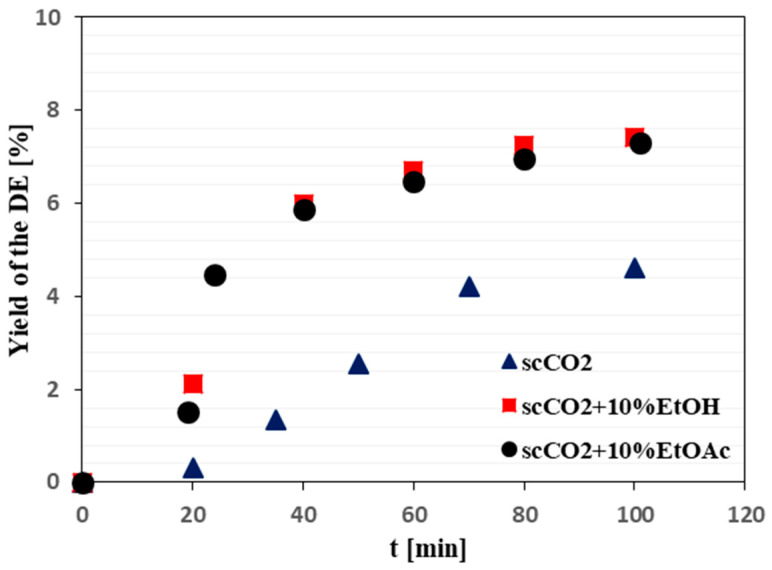
Effect of the SFE duration on the yield of dry *J. virginiana* leaf extract. Abbreviations: DE—dry extract; scCO_2_—supercritical carbon dioxide; EtOH—ethanol; EtOAc—ethyl acetate. The yield was determined as percentage of the mass of the dry extract, which was obtained during the SFE process, over the mass of the dry starting plant material.

The mass accumulation of the obtained dry extract over time was also monitored in order to determine the process duration necessary for the completion of SFE (Figure 6).

### 2.3. Accelerated Solvent Extraction of PPT from Juniper Leaves

The accelerated solvent extraction (ASE) of PPT from juniper leaves was carried out in ethanol or ethyl acetate at various temperatures (room temperature, 40 °C, 60 °C and 80 °C). The process was performed in three cycles (15 min each) and the pressure was set at 10.35 MPa (1500 psi). The effects of the solvent and temperature on the PPT recovery were analyzed (Table 5).

### 2.4. The UHPLC-HRMS Method for PPT Quantification in Juniper Leaf Extracts and Its Validation

**Figure 7:**
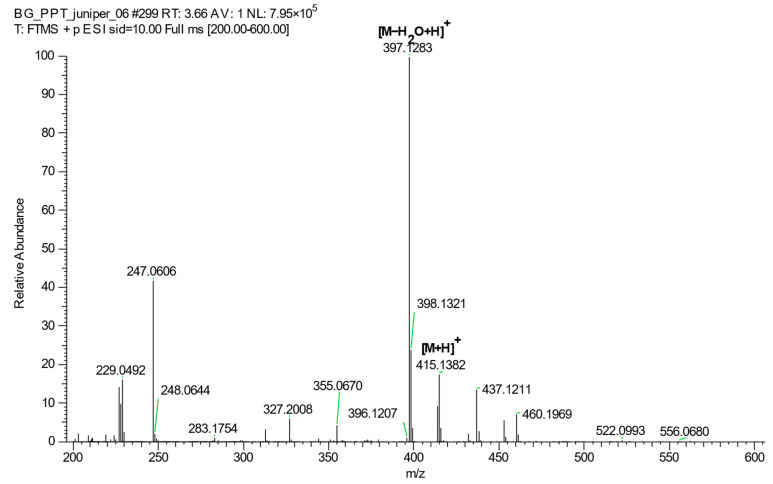
The full MS-SIM spectrum of podophyllotoxin.

The full MS-SIM (mass spectrometry-selected ion monitoring) spectrum of podophyllotoxin showed a protonated molecule [M + H]+ at m/z 415.1382 (Δppm = −1.39) and a product ion due to the neutral loss of water [M − H_2_O + H]+ at m/z 397.1283 (Δppm = 0.18) (Figure 7). The latter was the most intensive and characteristic peak of podophyllotoxin [25]; therefore, it was selected as a quantifier with a 5 ppm mass range window. Applying 10 eV in source collision-induced dissociation (CID) improved the intensity of the quantifier ion. In addition, to minimize the complexity of the matrix (presence of chlorophylls, etc.) each sample was subjected to solid-phase extraction (SPE) before dilution and injection.

The calibration curve of podophyllotoxin (PPT) was linear over the concentration range from 12.25 to 392 ng/mL and showed very good linear regression. The regression coefficient was R^2^ = 0.9996. The method showed that the LOD and LOQ were 0.32 ng/mL and 0.97 ng/mL, respectively (Table 6).

The accuracy of PPT quantification was checked at three different concentrations (49.00, 98.00 and 147.00 ng/mL).

The external standard showed overall recoveries ranging from 99.73% to 101.79%, with the relative standard deviation (RSD) ranging from 1.05% to 1.15% (Table 7).

The precision of the retention times was determined by analyzing the calibration sample during a single day and on three different days. The RSDs of the retention times of podophyllotoxin were 0.17 and 0.14 for intra-day and inter-day precision assays, respectively. Additionally, the external standard showed recoveries at 101.98% (for the intra-day precision assay) and 101.83% (for the inter-day precision assay) with RSDs at 1.56% and 1.02%, respectively (Table 8).

### 2.5. Seasonal and Storage Stability of the PPT in Juniper Leaves

- Seasonal stability of the PPT in juniper leaves

The seasonal stability of the PPT biosynthesis in *J. virginiana* was analyzed by detecting the PPT content in the leaf extracts, which were obtained from two individuals (female and male juniper representatives). For this experiment, juniper leaves were collected in the first decade of every month for two consequent years. (Table 9).

- Storage stability of the PPT content in juniper leaves

The stability of the plant material under different storage conditions was studied using *J. virginiana* leaves that were stored in a freezer (at −20 °C) or at room temperature. After storage of the juniper leaves for one year, the PPT content of the corresponding dry leaf extracts consisted of 21 ± 1 mg/g DE after storage at −20 °C (in a freezer) and 17 ± 1 mg/g DE after storage in the dark at room temperature.

## 3. Discussion

### 3.1. Influence of the Process Parameters on the PPT Recovery from Juniper Leaves by Extraction at Atmospheric Pressure

#### 3.1.1. Determination of Optimal Conditions for Conventional PPT Extraction from Juniper Leaves at Atmospheric Pressure

Previous studies reported various methods for optimization of the conditions for PPT delivery from junipers; however, the optimal conditions for PPT extraction from juniper leaves using an eco-friendly approach, which is feasible for the industrial production of this drug precursor, have not yet been established. A classical study was focused on the optimization of the extraction of PPT and other lignans from *J. bermudiana* L. leaves and found the following optimal extraction conditions: solvent methanol and a process duration of 5 h at 25 °C while stirring in a water bath [17]. This method was applied to several juniper species. The highest PPT content was determined in the leaves of *J. virginiana* (17.8 ± 1.3 mg/g DW (weight of the dry starting material)) and *J. bermudiana* (22.6 ± 0.5 mg/g DW). A recent study on the ultrasonic extraction of *J. sabina* leaves reported the following optimal conditions for PPT extraction: a liquid-to-solid ratio (LSR) of 40, 90% methanol, and an ultrasonic time of 7 min. The PPT content in *J. sabina* leaves was found to be 7.51 mg/g DW [26].

In the present study, the experiments for PPT extraction from juniper leaves at atmospheric pressure (shaker water bath) were carried out using solvents with different polarities. The analysis of the influence of the solvent type on the PPT recovery from juniper leaves showed that the best PPT content relative to the starting plant material was obtained in methanol or ethyl acetate (Table 1). On the other side, ethyl acetate extracted PPT more selectively and led to the PPT content having higher values in the final dry extract in comparison with alcohols (Figure 1). In our study, acetonitrile led to the best PPT content in the dry extract (Figure 1); however, it demonstrated a low yield of PPT recovered relative to the starting plant material (Table 1).

In addition, considering the potential practical application of the present results, ecological and safety criteria were imposed on the choice of the extraction solvent. Thus, it was taken into account that methanol can poison living beings via ingestion, leading to the destruction of the optic nerve and central nervous system. It can be metabolized into formaldehyde and formic acid, which are lethal to living cells [27]. Acetonitrile exhibits modest toxicity in humans (by inhalation and other effects) [28] and is prohibited as a component in cosmetic products [29].

On the other side, ethyl acetate has little toxicity and persists at low concentrations in fruits, wines, etc. Therefore, ethyl acetate was selected as an extraction solvent for the further optimization of the PPT content in the extracts.

The variation in the extraction temperature revealed that the increase in the temperature led to an increase in the yield of the final dry extract (Table 2), but the PPT content in the corresponding dry extracts decreased (Figure 2). It can be assumed that this molecule is thermally instable. Additionally, the solubility of other components increased, resulting in a lower PPT concentration. These opposite effects of the temperature on PPT recovery led to similar values of the PPT content relative to the weight of the starting plant material (Table 2). The highest concentrations of PPT in the dry juniper leaf extracts were obtained at room temperature (Figure 2), which was chosen for further extraction of PPT for the optimization process. The processing at room temperature also enables extraction at a lower cost without the need of heating or cooling.

The variation in the liquid-to-solid ratio LSR (*v*/*w*) when using the previously selected solvent ethyl acetate at room temperature revealed the effect of this parameter on the PPT concentration in the dry extract, as well as relative to the starting plant material (Table 3). The best results were obtained at an LSR of 10 (*v*/*w*). In addition, the increase of the solvent volume, resp. of a higher LSR, resulted in diluted extracts with a lower PPT concentration (Figure 3). Consequently, due to solvent consumption being lower and solvent regeneration being cheaper, an LSR of 10 (*v*/*w*) was selected for further PPT extraction optimization.

The analysis of the effect of the extraction time (Figure 4) with the previously selected conditions (solvent ethyl acetate, room temperature and an LSR of 10 (*v*/*w*)) showed that the extraction in ethyl acetate proceeds relatively quickly, and thus, a one hour process duration was sufficient for reaching the PPT equilibrium concentration (30 ± 1 mg/g DE, 3.4 ± 0.1 mg/g DW). In this study, the results of the PPT recovery from *J. virginiana* leaves were in agreement with previous studies of various *Juniperus* species, which found the PPT content to be most often in the range of about 1–7 mg/g DW [15,26], but extreme values (e.g., 23 mg/g DW in the leaves of endemic *J. bermudiana* L.) have been also reported [17].

#### 3.1.2. Correlation of Peleg’s Kinetic Modeling with PPT Extraction Duration

Peleg’s kinetic model is widely used for analysis of plant metabolite extraction [23]. In brief, Peleg’s equation reads:

(1) $$C\left(t\right)=C_{0}+\frac{t}{K_{1}t+K_{2}}$$

where *C*(*t*) is the concentration of PPT (mg/g DE) at any time *t* (h), *C*_0_ is the initial concentration of the extracted substance in the solvent at the moment *t* = 0, and *K*_1_ and *K*_2_ are Peleg’s constants.

The initial concentration of PPT is zero because a pure solvent is used in the beginning of the extraction experiment; thus, *C*_0_ = 0. Consequently, Equation (1) takes the form:

(2) $$C\left(t\right)=\frac{t}{K_{1}t+K_{2}}$$

Set in a linear form, Equation (2) becomes:

(3) $$\frac{t}{C\left(t\right)}=K_{1}t+K_{2}$$

It is seen that *K*_1_ and *K*_2_ represent the slope and the intercept of this straight line.

The extraction rate at any time *t* can be obtained by differentiation of Equation (2), where at the beginning of the process (*t* = 0) the differentiation equation is reduced to:

(4) $$\frac{dC\left(0\right)}{dt}=\frac{1}{K_{2}}=R_{0}$$

Therefore, the physical meaning of *K*_2_ is related to the initial extraction rate *R*_0_.

When *t* → ∞, i.e., at the equilibrium state, the term *K*_1_*t* takes much greater values than *K*_2_ (*K*_2_ can be neglected), and Equation (2) becomes:

(5) $$C{\left(t\right)}_{|t\to \infty }=C_{e}=\frac{1}{K_{1}}$$

Thus, the constant *K*_1_ is related to the equilibrium concentration *C_e_* of PPT.

**Figure 8:**
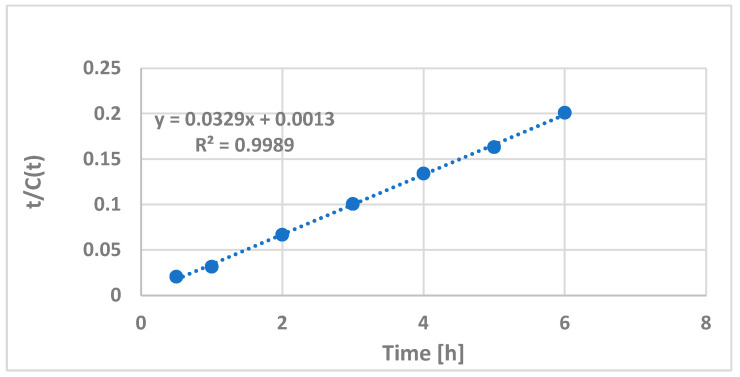
Extraction kinetics of PPT according to linearized Peleg’s Equation (3). Variable parameter—extraction time; constant parameters (previously selected conditions)—solvent ethyl acetate, room temperature and liquid-to-solid ratio of 10 (*v*/*w*). R—regression coefficient.

The linear form of Peleg’s Equation (3) with our experimental data is represented in Figure 8.

The resulting linear expression is:

t/C(t) = 0.0329t + 0.0013(6)

The coefficients *K*_1_ = 0.0329 and *K*_2_ = 0.0013 are used to calculate the equilibrium and rate constants, namely, the initial extraction rate *R*_0_ = 1/*K*_2_ = 769 (h^−1^) and the model equilibrium concentration *C_e_* = 1/*K*_1_ = 30 (mg/g DE). The latter is in rather good agreement with our experimental PPT equilibrium concentration (30 ± 1 mg/g DE). This PPT content of the DE corresponded to a PPT content of 3.4 ± 0.1 mg/g DW relative to the starting plant material.

In conclusion, a good correlation of Peleg’s equation curve with the presented experimental data was observed (Figure 4). It was confirmed that 1 h was sufficient to reach the pseudo-equilibrium concentration for ethyl acetate extraction of PPT from juniper leaves at room temperature and an LSR of 10 (*v*/*w*).

The 3D graph (Figure 5) covers the experimental data of time and temperature variation at the selected optimal liquid-to-solid ratio of 10. It visualizes the fast initial extraction rate, showing that there is no significant change of rate after one hour, and displays the maximum PPT concentration obtained by the ethyl acetate extraction of *J. virginiana* leaves at room temperature and a duration of one hour.

### 3.2. Effects of the Extraction Conditions on the PPT Recovery from Juniper Leaves by Supercritical Fluid Extraction (SFE)

Previous studies have shown that lignans can be effectively extracted from plants using supercritical carbon dioxide [12,30]. In the present study, the SFE method was employed as an ecological approach for the delivery of PPT from juniper leaves.

In the case of our study of *J. virginiana* leaves, which were used as an alternative source of PPT, the SFE was carried out at conditions corresponding to the supercritical state of the applied extraction fluids (neat scCO_2_ or scCO_2_, modified with ethanol or ethyl acetate): a pressure of 300 bar and temperature of 50 °C. The mass accumulation of the total dry extract during SFE was also monitored, and the results showed that 100 min were sufficient for completion of the process (Figure 6). The highest amount of PPT in mg per gram of the dry extract was obtained using scCO_2_ modified with ethanol or ethyl acetate (Table 4).

The PPT content in the dry extract obtained under supercritical conditions (42 ± 1 mg/g DE) using scCO2 modified with ethanol or ethyl acetate was higher than the corresponding PPT concentration obtained at normal pressure (30 ± 1 mg/g DE). On the other hand, the PPT recovery by SFE from the starting material was similar to the PPT content obtained at normal pressure (about 3 mg/g DW). These results reveal that SFE was a more selective method for PPT recovery than the conventional extraction at atmospheric pressure. Thus, the best conditions for PPT recovery from juniper leaves by SFE were found to include scCO_2_ modified with 10% ethanol or 10% ethyl acetate (Table 4).

### 3.3. Effect of the Process Conditions on the PPT Recovery from Juniper Leaves by Accelerated Solvent Extraction

The analysis of the effect of the solvent type and the temperature on the PPT recovery by ASE from juniper leaves showed that the best PPT content (45.5 ± 0.8 mg/g DE) in the final dry extract was obtained using ethyl acetate at room temperature. On the other hand, PPT recovery from the starting material was similar (about 3–4 mg/g DW) in ethanol or ethyl acetate due to higher dry extract yields in ethanol in comparison with the corresponding yields in ethyl acetate (Table 5). These results revealed ethyl acetate as a more selective solvent than ethanol for PPT recovery by ASE. Thus, solvent ethyl acetate and room temperature were selected as the optimal conditions for PPT recovery by ASE.

### 3.4. Seasonal and Storage Stability of PPT in Juniper Leaves

Previous experiments have revealed that the production of lignans in some plant species may be subjected to seasonal changes [31]. Other studies have found stable seasonal PPT biosynthesis in junipers [17].

The results of the present study determined that the PPT content in *J. virginiana* leaves was maintained at similar levels throughout the year; however, highest PPT concentrations were detected in the leaf extracts from January to April. Thus, January to April is the best period for raw material collection (Table 9).

In addition, a high stability (for at least one year) of PPT was observed in juniper leaves that were stored at room temperature (in the dark) or in a freezer. This result was assumed to be due to the stabilization of the PPT molecule as a glycoside in the plant material, the presence of natural antioxidants, etc.

The results regarding the seasonal and storage stability of the plant material suggest that evergreen junipers with efficient PPT biosynthesis, such as *J. virginiana*, are perspective natural raw materials for the delivery of the drug precursor PPT throughout the year.

## 4. Materials and Methods

### 4.1. Chemicals and Reagents

Podophyllotoxin (standard compound, ≥98%) was obtained from Sigma-Aldrich Chemie GmbH (Steinheim, Germany). Extraction solvents (pure analysis grade) were purchased as follows: methanol, ethanol, *i-*propanol, acetone and acetonitrile from Honeywell Riedel-de Haën GmbH (Seelze, Germany); ethyl acetate from JLS-Chemie Handels GmbH (Wiener Neustadt, Austria); *n-*butanol from Sigma-Aldrich Chemie GmbH (Steinheim, Germany); methyl ethyl ketone (butanone) from Merck (Darmstadt, Germany); and tetrahydrofuran from Fisher Chemical (Geel, Belgium). Ethanol and ethyl acetate for accelerated solvent extraction were purchased from Avantor Performance Materials (Gliwice, Poland). LC-grade acetonitrile was purchased from Sigma-Aldrich Fine Chemicals (Saint Louis, MO, USA). Formic acid was purchased from Möller Chemie GmbH & Co. KG (Steinfurt, Germany). LC-grade water was prepared using a Millipore Direct-Q3 purification system (Bedford, MA, USA). The solid phase extraction (SPE) was carried out on Phenomenex (Torrance, CA, USA) Strata^®^ C18-E (55 µm, 70 Å, 200 mg and 3 mL) cartridges that were activated prior to each procedure according to the manufacturer’s protocol.

### 4.2. Plant Material

Extraction optimization experiments were carried out using *Juniperus virginiana* (male representative) leaves. The plant material for the extraction optimization experiments was collected in February 2020 from Sofia, Bulgaria (42°43′25.9″ N; 23°18′10.6″ E, 550 m a.s.l.). A voucher specimen SOM 178263, authenticated by A. N. Tashev, was deposited in the Herbarium (SOM) of the Institute of Biodiversity and Ecosystem Research, Bulgarian Academy of Sciences (IBER-BAS).

### 4.3. Extraction Procedures

#### 4.3.1. Extraction at Atmospheric Pressure

The plant material was dried at room temperature until a constant weight was reached, and then kept in closed vessels in a freezer (at −20 °C). For extraction optimization experiments, *J. virginiana* (male representative) leaves (2 g) were ground and mixed with the corresponding solvent (at various liquid/solid ratios) in an Erlenmeyer flask with a stopper. The suspension was stirred in a shaker water bath at different temperatures and extraction times. The mixture was filtered, and the corresponding extract was collected and kept in a freezer until analyses. The results were the average of at least 2 experiments. Before UHPLC-MS, the extracts were sonicated for 3 min at room temperature in order to dissolve crystals appearing in the solutions during refrigeration.

The seasonal stability of PPT biosynthesis in junipers and the stability of the plant material under different storage conditions were analyzed using extracts, obtained by a previous modified method [16] using a single extraction procedure for 5 h in 80% (*v/v*) methanol.

#### 4.3.2. Supercritical Fluid Extraction (SFE)

The SFE experiments were carried out using an SFT-110-XW apparatus (Supercritical Fluid Technologies Inc., Newark, DE, USA), as was described previously [32]. In brief, 5 g of dry ground *J. virginiana* leaves were subjected to SFE at 50 °C, a pressure of 300 bar (30 MPa) and a process duration of up to 100 min. The following extraction fluids were used: neat scCO_2_ or scCO_2_ with co-solvents ethanol (10%) and ethyl acetate (10%). In all experiments, the final fluid flow was maintained at 1.9 × 10^−3^ kg·min^−1^. The extracts were collected in ice-cooled glass vials at ambient pressure. Co-solvents were evaporated using the HEI-Vap Value vacuum evaporator (Heidolph Instruments GmbH & Co. KG, Schwabach, Germany). After that, the extracts were freeze-dried (24 h, −55 °C, 0.05 mbar) on an Alpha 1-2 LDplus freeze-dryer (Martin Christ Gefriertrocknungsanlagen GmbH, Osterode, Germany) and kept in the freezer until UHPLC analyses. The SFE experiments were performed in duplicate.

#### 4.3.3. Accelerated Solvent Extraction (ASE)

The extracts were obtained using the accelerated solvent extraction system (ASE 150 extractor; Dionex Corporation; Sunnyvale, CA, USA). The homogenized dry plant material (1 g) was mixed (1:1, *v*/*v*) with diatomaceous earth and packed into a 5 mL extraction cell. Three extraction cycles (15 min each) were performed using a pressure of 10.35 MPa (1500 psi), and solvent ethanol or ethyl acetate at various temperatures (20 °C, 40 °C, 60 °C or 80 °C). Eluates were filtered, filled up to the same volume with the proper solvent and kept in the freezer until UHPLC analyses. The ASE experiments were carried out in duplicate.

### 4.4. Ultra-High-Performance Liquid Chromatography Coupled to High-Resolution Mass Spectrometry (UHPLC-HRMS) Analysis

UHPLC-HRMS analysis was performed using a Thermo Scientific Dionex Ultimate 3000 RSLC (Germering, Germany) consisting of the 6-channel degasser SRD-3600, HPG-3400RS high-pressure gradient pump, WPS-3000TRS autosampler, and TCC3000RS column compartment coupled with the Thermo Scientific Q Exactive Plus (Bremen, Germany) mass spectrometer. The separations were achieved with Thermo Fisher Scientific’s (Waltham, MA, USA) Hypersil GOLD C18 Selectivity column (1.9 μm, 2.1 × 50 mm) equipped with Waters (USA) VanGuard C18 precolumn (2.7 µm, 2.1 × 5 mm) at 40 °C. Each chromatographic run was carried out with a binary mobile phase consisting of water containing 0.1% (*v*/*v*) formic acid (A) and acetonitrile with 0.1% (*v*/*v*) formic acid (B). A gradient program was used as follows: 0–4 min, 27% B; 4–4.5 min, 27–35% B; 4.5–6 min, 35% B; 6–8 min, 35–95% B; 8–10 min, 95% B. The column was equilibrated under the initial conditions for 3 min before each injection. The flow rate and the sample injection volume were 0.3 mL·min^−1^ and 2 µL, respectively. Operating conditions for the HESI source used in a positive ionization mode were as follows: a spray voltage of +3.5 kV, capillary and probe heater temperature of 320 °C, sheath gas flow rate of 36 a.u., auxiliary gas flow of 11 a.u., spare gas flow of 1 a.u. (a.u. refers to arbitrary values set by the Exactive Tune software ver. 2.8 SP1) and S-Lens RF level of 50.00. Nitrogen was used for sample nebulization and as collision gas in the HCD cell. The full MS-SIM mode was used as an MS experiment where the resolution, automatic gain control (AGC) target, maximum inject time (IT) and mass range were 70,000 (at *m*/*z* 200), 3 × 10^6^, 200 ms and *m*/*z* 200–600, respectively. The in-source CID was set to 10.0 eV. The product ion was at *m*/*z* 397.1282, with a 5.0 ppm isolation window used as a quantifier. Xcalibur software ver. 4.0 was used for data acquisition and processing.

### 4.5. Sample Preparation Prior to UHPLC-HRMS Analysis

In total, 5 milligrams of each extract were dissolved in 800 µL of MeOH using an ultrasonic bath, and then 200 µL of H_2_O was added. Each of the resulting solutions was passed through an SPE cartridge, followed by four-fold washing with 1 mL of 80 vol.% MeOH. Finally, the solutions were diluted to 10 mL with 80 vol.% MeOH. Each solution was diluted to a final concentration of 2 µg/mL.

### 4.6. Validation of the UHPLC-HRMS Method for PPT Quantification in Juniper Leaf Extracts

The quantification of podophyllotoxin was carried out using the external standard method. External standard calibration was established on six data points covering the concentration range of 12.25–392 ng/mL for podophyllotoxin.

The limit of detection (LOD) of the analytical procedure was determined as the lowest analytical concentration at which an analyte(s) could be detected. Typically, peak heights are two or three times the noise level. The quantitation limit (LOQ) was also the lowest concentration at the level of the analyte that can be quantitated with acceptable precision, requiring peak heights 10 to 20 times higher than the baseline noise. This signal-to-noise ratio is a good rule of thumb. Limits of detection (LODs) were calculated according to the expression 3.3 σ/S, where σ was the standard deviation of the response and S the slope of the calibration curve. Limits of quantification (LOQs) were established from the expression 10 σ/S [33,34].

Accuracy is the closeness of the analytical results obtained by the analyses to the true values, and usually it is presented as a percent of the nominal [33,34]. The accuracy of analytes was evaluated by applying the entire extraction procedure to a control plant matrix that had been spiked with a standard solution of podophyllotoxin at three concentrations. The accuracy data were recorded as the percent recovered from the spiked concentration with relative standard deviations. Each solution was tested in triplicate.

The precision of an analytical method is the amount of variation in the results obtained from multiple analyses of the homogeneous samples. Intra-day precision (repeatability) defines the precision obtained using the same operating conditions over a designated short period (typically ≤1 day). Inter-day precision (intermediate precision) defines the precision obtained using the same operating conditions, typically within the same laboratory, over a designated period (typically ≥1 day) [33,35]. The intra-day and inter-day precision were determined by analyzing the calibration samples during a single day and on three different days, respectively. The intra-day variation was determined by analyzing the nine replicates on the same day and the inter-day variation was determined over three consecutive days. The retention times (RTs) and recovery were obtained for the assayed compounds. The relative standard deviation (RSD) was taken as a measure of precision.

## 5. Conclusions

According to previous studies [15,16], several juniper species were found to efficiently produce podophyllotoxin, and their leaf extracts were considered as potential alternative sources of this drug precursor for use in the pharmacy. The present research employed one of them (*Juniperus virginiana* leaves) as an experimental model for identification of optimal conditions for PPT recovery using various extraction methods. The PPT content in *J. virginiana* leaves, used as the experimental model in the present study, was similar to that found in previous studies of various *Juniperus* species, which registered the PPT content to be most often in the range of 1–7 mg/g DW [15,26]. To our knowledge, the present research is the first PPT extraction optimization study of juniper species that is focused on ecological solvents in view of presumable industrial applications.

The optimal podophyllotoxin content of the dry extract (in the range of 30 mg/g DE, which corresponded to 3 mg/g DW relative to the dry starting material) was determined at normal pressure at the following extraction conditions: solvent ethyl acetate, room temperature, liquid-to-solid ratio of 10 *v*/*w* and a 1 h process duration applying agitation. The experimental results and the theoretical predictions about the optimal process parameters for PPT atmospheric extraction were correlated and confirmed by Peleg’s kinetic modeling.

However, in comparison with normal pressure extraction, a higher PPT content in the dry extract (about 42 mg/g DE) was obtained at supercritical conditions (300 bar, 50 °C and 100 min duration) using scCO_2_ modified with ethanol (10%) or ethyl acetate (10%). On the other hand, PPT recovery by SFE or at normal pressure in relation to the starting material was similar (about 3 mg/g DW), which revealed that SFE was a more selective method than normal pressure extraction.

The other high pressure extraction method, accelerated solvent extraction, showed the highest PPT content in the dry extract (about 45 mg/g DE) at room temperature using ethyl acetate as a solvent. The comparison of the PPT content in the final DE using solvent ethanol or ethyl acetate with similar values for PPT recovery (3–4 mg/g DW) in relation to the starting material revealed ethyl acetate as being a more selective solvent than ethanol.

In conclusion, the extracts obtained by the three studied methods were about 10–15 times more concentrated regarding PPT than the starting raw material. PPT extraction under normal atmospheric pressure has the advantage of using a simple and cheap technique, which is feasible for prospective industrial producers. However, the high-pressure extraction methods ASE and SFE led to the highest PPT content in the final dry extracts and were revealed to be the best methods for PPT delivery from juniper leaves.

The study of seasonal PPT biosynthesis showed that juniper leaves can be used throughout the year; however, January to April is the best period for raw material collection. Juniper leaves showed stable PPT content for at least one year at room temperature or in a freezer.

The prospects for the further development of the present research include the identification of other bioactive substances in juniper leaves in addition to the previously found anticancer lignans ([15,16], etc.), as well as the improvement of the extraction purification process by treatment with a non-polar solvent for removal of lipophilic components (terpenes, phytosterols, waxes, carotenoids, lipids, chlorophylls, etc.) from the obtained liquid extracts. The botanical selection of juniper representatives for use in the pharmacy with a higher efficiency of PPT biosynthesis is envisaged in the future.

The presented optimized processing for PPT recovery from juniper leaves is expected to have potential applications as an appropriate alternative technological approach for the production of this drug precursor for use in the pharmacy.

## Figures and Tables

**Table 1:** Effect of the solvent type on the podophyllotoxin concentration relative to the weight of the starting plant material, *J. virginiana* leaves.

Solvent	Water	MeOH	EtOH	*i-*PrOH	*n-*BuOH	THF	MeCN	EtOAc	Acetone	MEK
PPT (mg/g DW)	0.8 ± 0.2	4.1 ± 0.2	2.9 ± 0.2	1.9 ± 0.2	2.1 ± 0.1	2.7 ± 0.1	2.4 ± 0.3	3.5 ± 0.1	2.2 ± 0.2	2.0 ± 0.3

Legend: Extraction conditions—liquid-to-solid ratio of 10 (*v*/*w*), process duration of 5 h and room temperature. Abbreviations: PPT—podophyllotoxin; DW—weight of the dry starting plant material; MeOH—methanol; EtOH—ethanol; *i-*PrOH—*i-*propanol; *n-*BuOH—*n-*butanol; THF—tetrahydrofuran; MeCN—acetonitrile; EtOAc—ethyl acetate; MEK—methyl ethyl ketone (2-butanone).

**Table 2:** Effect of the temperature on the total yield of the juniper leaf extracts and on the PPT recovery relative to the weight of the starting plant material.

Temperature	20 °C	40 °C	50 °C	60 °C	70 °C
DE yield (%)	11.0 ± 0.7	12.0 ± 0.6	13.0 ± 0.9	14.0 ± 0.6	15.2 ± 0.5
PPT (mg/g DW)	3.3 ± 0.2	3.1 ± 0.2	3.2 ± 0.2	3.4 ± 0.1	3.4 ± 0.1

Legend: The yield of the DE was calculated as percentage of the weight of the dry extract over the weight of the dry starting plant material. Other extraction parameters—solvent of ethyl acetate, liquid-to-solid ratio of 10 (*v*/*w*) and process duration of 5 h. Abbreviations: PPT—podophyllotoxin; DW—weight of the dry starting plant material; DE—dry extract.

**Table 3:** Effect of the liquid-to-solid ratio on the PPT concentration relative to the obtained dry *J. virginiana* leaf extracts and to the weight of starting plant material.

LSR (*v*/*w*)	10	20	30	40
PPT (mg/g DE)	30 ± 3	24 ± 2	25 ± 1	25 ± 1
PPT (mg/g DW)	3.1 ± 0.2	2.6 ± 0.1	2.7 ± 0.1	2.7 ± 0.1

Legend: Variable parameter—liquid-to-solid ratio (*v*/*w*); constant parameters—process duration (5 h), previously selected solvent (ethyl acetate) and room temperature (20 °C). Abbreviations: PPT—podophyllotoxin; DE—dry extract; DW—weight of the dry starting plant material; LSR—liquid-to-solid ratio.

**Table 4:** Effect of the solvent on the PPT recovery by supercritical fluid extraction of *J. virginiana* leaves.

SFE Solvent	scCO_2_	scCO_2_ + 10% EtOH	scCO_2_ + 10% EtOAc
PPT (mg/g DE)	24 ± 1	42 ± 1	42 ± 1
PPT (mg/g DW)	1.1 ± 0.1	3.1 ± 0.1	3.1 ± 0.1

Legend: Variable parameter—supercritical fluid; constant conditions—temperature of 50 °C, pressure of 300 bar and duration of 100 min. Abbreviations: PPT—podophyllotoxin; DE—dry extract; DW—weight of the dry initial plant material; scCO_2_—supercritical carbon dioxide; EtOH—ethanol; EtOAc—ethyl acetate.

**Table 5:** Effects of the solvent and temperature on the PPT recovery by accelerated solvent extraction of *J. virginiana* leaves.

	Conditions	EtOH 20 °C	EtOH 40 °C	EtOH 60 °C	EtOH 80 °C	EtOAc 20 °C	EtOAc 40 °C	EtOAc 60 °C	EtOAc 80 °C
Yields									
DE Yield (%)		17.5 ± 0.2	21.7 ± 0.2	25.0 ± 1.0	28.5 ± 1.1	6.5 ± 0.2	8.4 ± 0.1	10.6 ± 0.1	12.3 ± 0.7
PPT (mg/g DE)		16.3 ± 0.2	15.4 ± 0.8	15.0 ± 1.6	12.8 ± 0.3	45.5 ± 0.8	39.5 ± 1.9	32.9 ± 0.8	30.0 ± 0.2
PPT (mg/g DW)		2.9 ± 0.1	3.3 ± 0.1	3.7 ± 0.2	3.6 ± 0.2	3.0 ± 0.1	3.3 ± 0.2	3.5 ± 0.1	3.7 ± 0.2

Legend: Variable ASE conditions—solvent and temperature; constant parameters of the process—pressure of 10.35 MPa and 3 extraction cycles (15 min each). Abbreviations: PPT—podophyllotoxin (mg/g DE); DE—dry extract; DW—weight of the dried initial plant material.

**Table 6:** Linearity parameters of the PPT calibration curve and limits of detection and quantification.

External Standard	Linear Range (ng/mL)	Regression Equation	R^2^	LOD (ng/mL)	LOQ (ng/mL)
podophyllotoxin	12.25–392	Y = 228,429 + 198,179 × X	0.9996	0.32	0.97

Abbreviations: LOD—limit of detection; LOQ—limit of quantification.

**Table 7:** Accuracy of the UHPLC-HRMS method.

External Standard	Added (ng/mL)	Found ^1^ (ng/mL)	Recovery ^1^ (%)	RSD (%)
	49.00	49.41 ± 0.52	100.83 ± 1.06	1.05
podophyllotoxin	98.00	97.73 ± 1.12	99.73 ± 1.15	1.15
	147.00	149.64 ± 1.69	101.79 ± 1.15	1.13

^1^ Values are represented as the mean ± SD (*n* = 3). Each solution was tested in triplicate. Abbreviation: RSD—relative standard deviation.

**Table 8:** Evaluation of intra-day (repeatability) and inter-day (intermediate precision) precision of the UHPLC-HRMS method applied to podophyllotoxin quantification.

Precision Type	RT ± SD (min)	RSD (%)	Recovery ± SD (%)	RSD (%)
Intra-day	3.63 ± 0.006	0.17	101.98 ± 1.59	1.56
Inter-day	3.62 ± 0.005	0.14	101.83 ± 1.04	1.02

Abbreviations: RT—retention time; SD—standard deviation; RSD—relative standard deviation.

**Table 9:** Seasonal stability of the PPT content in *J. virginiana* leaf extracts.

Month	Jan	Feb	Mar	Apr	May	June	July	Aug	Sep	Oct	Nov	Dec
PPT	22 ± 1	20 ± 2	19 ± 3	23 ± 1	17 ± 1	16 ± 1	17 ± 2	16 ± 3	16 ± 1	17 ± 1	18 ± 2	18 ± 1

Legend: PPT—podophyllotoxin (mg/g DE); DE—dry extract. The values are average ± standard deviations of the PPT content in the leaf extracts obtained from two juniper individuals (female and male) and analyzed for two consequent years.

## Data Availability

Data is contained within the article.
